# Plasmon-Enhanced Photocurrent using Gold Nanoparticles on a Three-Dimensional TiO_2_ Nanowire-Web Electrode

**DOI:** 10.1038/srep42524

**Published:** 2017-02-10

**Authors:** Yin-Cheng Yen, Jau-An Chen, Sheng Ou, Yi-Shin Chen, Kuan-Jiuh Lin

**Affiliations:** 1 Department of Chemistry, National Chung Hsing University, Taichung 40227, Taiwan, Republic of China

## Abstract

In this study, an anatase/rutile mixed-phase titanium dioxide (TiO_2_) hierarchical network deposited with Au nanoparticles (Au/TiO_2_ ARHN) was synthesized using a facile hydrothermal method followed by a simple calcination step. Such a unique structure was designed for improving the light harvest, charge transportation/separation, and the performance of photo-electro-chemical (PEC) cells. The properties of the as-synthesized Au/TiO_2_ ARHN in PEC cells were investigated by electrochemical measurements using a three-electrode system in a 1 M NaOH electrolyte. Remarkably, a 4.5-folds enhancement of the photocurrent for Au/TiO_2_ ARHN was observed as compared to that for TiO_2_ nanowire (NW), under AM1.5G solar illumination, suggesting its potential application in PEC cells. A mechanism has been proposed to explain the high photocurrent of Au/TiO_2_ ARHN in PEC water splitting.

Honda and Fujishima elucidated the possibility of water splitting using TiO_2_ as electrode^1^. Since then, various TiO_2_ nano-architectonic topographies have been greatly desired for enhancing the performance of PEC cells^2^,^3^,^4^,^5^,^6^,^7^,^8^. In general, a predominant PEC cell relies on two factors: the efficient usage of solar energy and the instant transportation/separation of charges^9^. Hence, the development of nano-sized photo-active semiconductors to satisfy the requirements has been a long-standing objective in the research of PEC cells, especially for one-dimensional TiO_2_ due to its superior charge transport property^10^. To date, many hierarchical TiO_2_ nanostructures based on nanowires (NWs) and nanotubes (NTs) have been synthesized for enhanced photo-electric efficiency in solar energy harvesting, conversion, and pollutant purification^11^,^12^,^13^. Such a heterojunction nanostructure would stagger energy levels and scatter incident light to enlarge light absorption in the UV region^14^. However, the strategies for fabricating hierarchical TiO_2_ nanostructures have the disadvantages of an extremely time-consuming process, specific/highly expensive fabricating apparatus and back-side illumination, thus making it economically non-competitive^15^,^16^,^17^,^18^,^19^,^20^,^21^,^22^,^23^,^24^,^25^.

Recently, to expand the TiO_2_ optical adsorption spectrum from the UV into the visible region, plasmonic electrodes composed of Au/TiO_2_ nano-architectonic topographies have been developed with localized surface plasmon resonance (LSPR) property^26^,^27^,^28^,^29^,^30^,^31^,^32^,^33^,^34^,^35^. However, most of these reports focus on the discussion of the relationship between particle size/shape/distance/concentration and the photo-electrochemical performance^28^,^29^,^30^,^32^,^33^,^34^,^35^. Especially, one novel example of demonstrating the influence of TiO_2_ nanostructure on the LSPR property was reported by Wang *et al*.^36^. An Au/TiO_2_/Au nanostructure with a 5-nm-thick TiO_2_ middle layer was synthesized which resulted in a maximum 38-fold enhancement of the electric field density of LSPR and about 3-fold improvement of the photocurrent in a wavelength range of 400–650 nm. The enhanced performance is mainly arising from the thickness of TiO_2_ satisfying the requirement for generating the coupling effect between the oppositely aligned and nearly touching Au NPs on TiO_2_ nanosheet. However, the longer time (4 days) required to synthesize the TiO_2_ nanostructures and the non-transparency of the Au/TiO_2_/Au film limit their application. Furthermore, there is limited knowledge on how to design and synthesize a TiO_2_ nanostructured film on a transparent substrate by a simple yet effective method. Therefore, the aim of this study is to provide a novel strategy for building a new TiO_2_ nanostructure to intensify the coupling effect between Au NPs that significantly enhances the photoelectric conversion.

Herein, a three-dimensional (3D) web constructed by Au plasmonic NPs on TiO_2_ anatase/rutile hierarchical network (Au/TiO_2_ ARHN) is proposed, which is schematically shown in Fig. 1. In order to strengthen the electromagnetic coupling of the Au NPs, the solid support―TiO_2_ NWs connected with TiO_2_ threads― was synthesized by a two-step hydrothermal process. When tested in the PEC experiment, the TiO_2_ ARHN and Au/TiO_2_ ARHN exhibited 1.5 times and 4.5 times higher photocurrent than TiO_2_ NWs.

## Results and Discussion

**Figure 2:**
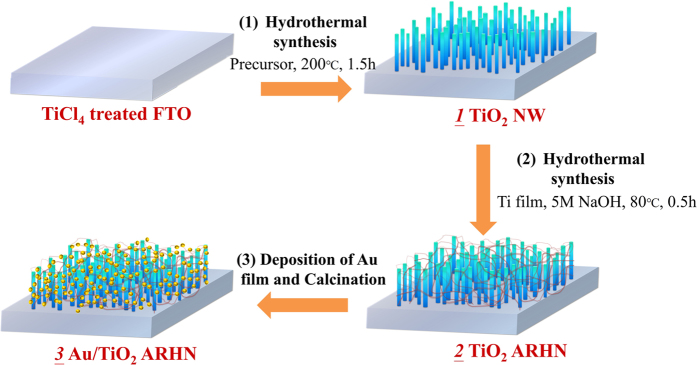
Schematic description of the synthesis process of TiO_2_ NWs, ARHN and Au/TiO_2_ ARHN on FTO substrates.

**Figure 3:**
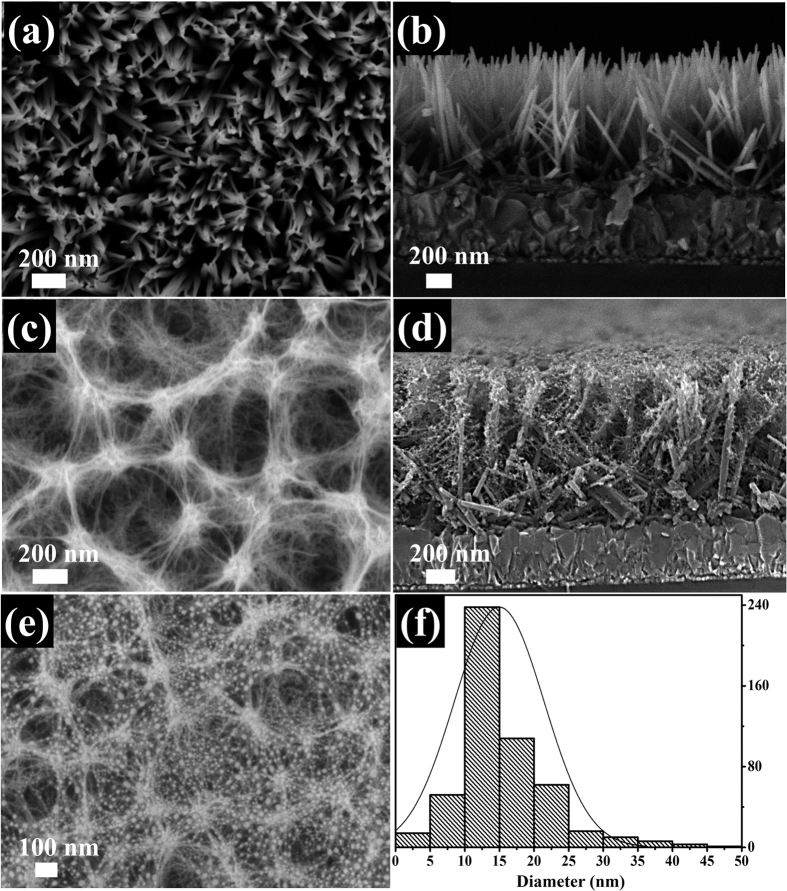
FESEM images. (**a**,**c**,**e**) top images of top TiO_2_ NW, TiO_2_ ARHN and Au/TiO_2_ ARHN, respectively; (**b**,**d**) cross-sectional images of TiO_2_ NW and TiO_2_ ARHN; (**f**) the size distribution of Au NPs.

Figure 2 schematically depicts the three-step fabrication process of reproducible Au/TiO_2_ ARHN. Figure 3a represents a top-view SEM image of the TiO_2_ NWs. The light-gray needle-like regions in the SEM image represent the TiO_2_ NWs and the dark regions are the underlying FTO substrate. A cross-sectional SEM image of the NWs (Fig. 3b) shows that the thickness of TiO_2_ layer is ~1 μm and the NWs have an average diameter of 40 nm. XRD patterns of the TiO_2_ NWs show predominantly rutile phase with preferential orientation of (110) (Supplementary Fig. 1). The top-view and cross-sectional SEM image of the TiO_2_ ARHN (Fig. 3c,d) shows that the TiO_2_ threads are bridged with the TiO_2_ NWs to form a 3D hierarchical network. Nest-like porous cavities with diameters of a few hundred nanometers are clearly observed and the diameters of the threads are ~10 nm. The XRD patterns and Raman spectra show that the threads belong to the anatase phase (Supplementary Figs 2 and 3). The top-view SEM image of the Au/TiO_2_ ARHN is shown in Fig. 3e. The white dot regions in the SEM represent the Au NPs. The size distribution histograms of Au NPs show an average particle size of 15 nm (Fig. 3f).

To investigate the formation mechanism of TiO_2_ ARHN, a series of experiments were performed. Firstly, we failed to obtain TiO_2_ ARHN without TiCl_4_ treatment. We found that TiO_2_ threads cannot grow on TiO_2_ NWs with a smooth surface. Secondly, in the absence of the Ti film in step 2 (in Fig. 2), only TiO_2_ NWs were observed. These findings suggest that both TiCl_4_ treatment and the formation of the Ti layer for the formation of TiO_2_ ARHN are indispensable. Therefore, we propose that small TiO_2_ seed crystals grow on the surface of TiO_2_ NWs after TiCl_4_ treatment, which leads to a rough surface for the growth of TiO_2_ threads. Moreover, during alkali hydrothermal process, the Ti layers can generate large amounts of Ti-containing species^37^,^38^ as precursors that can deposit on the TiO_2_ seeds and produce a network structure.

**Figure 4:**
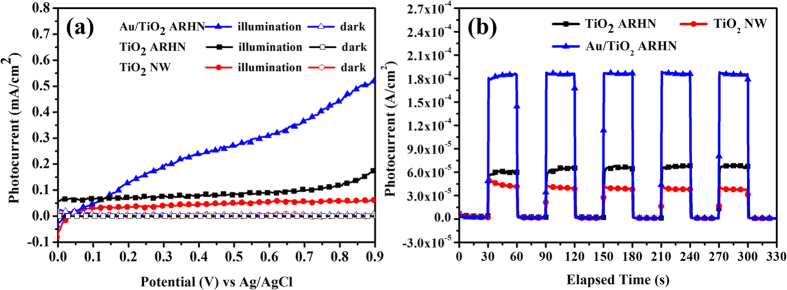
(**a**) Linear sweep voltammograms and (**b**) amperometric I―t curves of TiO_2_ NW, TiO_2_ ARHN and Au/TiO_2_ ARHN photoelectrode.

To evaluate the enhanced PEC performance of the designed Au/TiO_2_ ARHN, the linear sweep voltammograms and the photocurrent-versus-time (I-t curve) of TiO_2_ NW and TiO_2_ ARHN with/without Au NPs were conducted under AM 1.5 G simulated solar illumination, as shown in Fig. 4. The measured photocurrent was normalized to the sample area to obtain the photocurrent density for comparison. As presented in Fig. 4a, the TiO_2_ NW electrode produced a photocurrent density of 4 × 10^−5^ A cm^−2^ at 0.23 V *vs*. Ag/AgCl, which is the potential often chosen as a metric to evaluate the performance of photoanodes as it corresponds to the water oxidation potential^4^. The low photocurrent density is attributed to the limit of wide band-gap characteristics of TiO_2_ (3.2 eV for anatase^39^ and 3.0 eV for rutile^40^), due to which only UV light can be used in the PEC water splitting system. The photocurrent was enhanced for the TiO_2_ ARHN (6 × 10^−5^ A cm^−2^) when compared with TiO_2_ NW, with an enhancement factor of 1.5. As expected, a significant photocurrent density enhancement was clearly observed on the Au/TiO_2_ ARHN, having a photocurrent density of 1.8 × 10^−4^ A cm^−2^. As compared to TiO_2_ NW, a photocurrent enhancement higher than 4.5 times was achieved. From Fig. 4b, all electrode represent a good reproducibility and stability as the illumination was turned on and off. Furthermore, the sharp spike in the photocurrent during the on/off illumination cycles demonstrates the predominant transport of photogenerated electrons in the designed TiO_2_ structure^41^.

**Figure 5:**
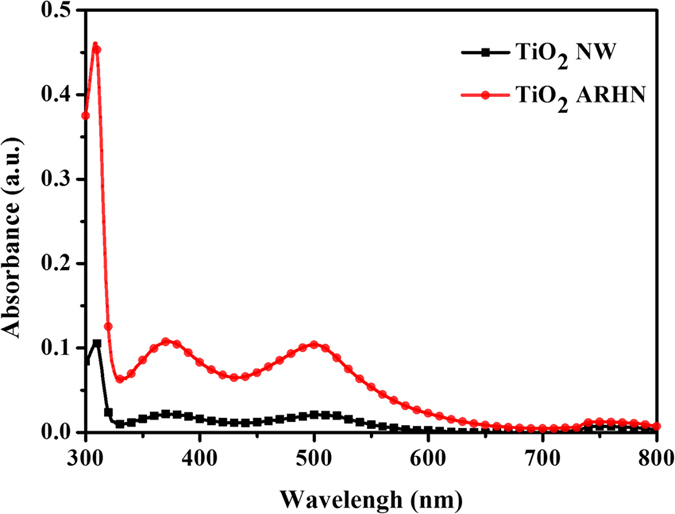
UV-Vis spectra of desorbed N719 solution.

We suggest the enhanced photocurrent of TiO_2_ ARHN electrode could be attributed to better photocatalytic activity, due to increased surface area, or better light harvest efficiency, due to the hierarchical network structure. Therefore, the dye absorption/desorption experiment and UV-visible spectrum measurement were perform to verify it. Here, dye N719 was choose as an adsorbate to execute dye absorption/desorption experiment due to it could be monolayer absorbed on the surface of TiO_2_. Therefore, we can evaluate the related surface area via measuring the absorption of N719 dye which detach from TiO_2_ structure. As shown in Fig. 5, there are three absorbed peak of N719 located on 310 nm, 370 nm and 505 nm, respectively^42^. It is observed that the absorption of detached N719 solution based on TiO_2_ ARHN is obviously large than TiO_2_ NW on entire spectrum. It represents the surface area of TiO_2_ ARHN is related large than TiO_2_ NW due to there are more dye absorbed on TiO_2_ ARHN. Based on the result, we make a sure that the high surface area of TiO_2_ ARHN is a reasonable reason which bring to a high photo activity on PEC measurement.

**Figure 6:**
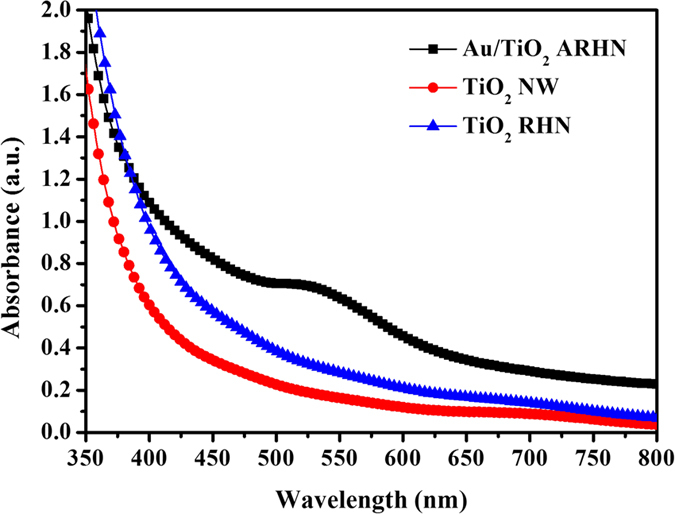
UV-Vis spectra of TiO_2_ NW, TiO_2_ ARHN and Au/TiO_2_ ARHN.

Furthermore, the UV-visible absorption spectra of the TiO_2_ NW and TiO_2_ ARHN with/without Au NPs are shown in Fig. 6. TiO_2_ NW exhibits a stronger absorption at the wavelengths below 400 nm due to electron transitions of TiO_2_ from the valence band to the conduction band. In addition, the absorption spectra of TiO_2_ ARHN showed an enhanced absorption in the entire spectral range as compared with TiO_2_ NW, which is attributed to the scattering effect in the ARHN structure; this also explains the increased photocurrent in the TiO_2_ ARHN. With deposited Au NPs, the absorption show a significantly enhancement on visible range which is driven by the LSPR absorption. From incident photon-to-electron conversion efficiency (IPCE) measurement (Supplementary Fig. 4), it demonstrates that such an absorption successfully boosts the PEC performance in the region from 400 nm to 700 nm.

**Figure 7:**
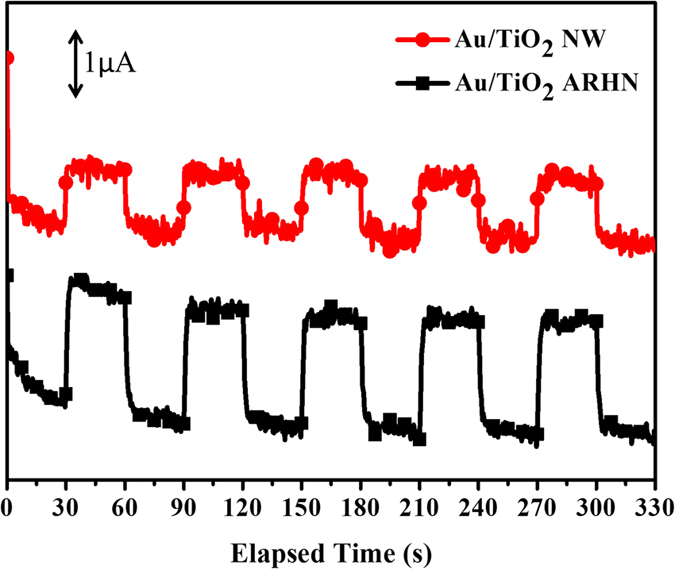
I − t curves collected at 0 V versus Ag/AgCl for Au/TiO_2_ NW and Au/TiO_2_ ARHN electrode under visible light illumination (using a cutoff filter with a wavelength of 400 nm).

In this work, a LSPR peak for the Au NPs with average size of 15 nm centered at around 540 nm. For Au NPs of size 10–20 nm, the absorption peak of plasmon resonance is usually located at 520–525 nm^43^,^44^. A redshift of 15–20 nm of the plasmon resonance peak was observed as compared to previous reports. This may be attributed to the TiO_2_ changing the surrounding dielectric property of Au NPs (Au NPs well deposited and in contact withTiO_2_ surface) and the enhancement of electromagnetic field of LSPR^30^,^45^,^46^. It has been reported that the high electromagnetic field of LSPR and strong coupling between Au NPs and TiO_2_ will benefit the plasmon-induced charge transportation and separation, enabling SPR-enhanced photocatalysis. Typically, the LSPR-induced charge separation at the interface between the Au NPs and TiO_2_ can occur by transferring the energy contained in the oscillating electrons or local plasmonic field from Au NPs to TiO_2_ through direct electron transfer, also known as hot electron injection^47^,^48^. Higher electromagnetic field generates more hot electron^49^,^50^. In order to verify our assumption, the design of TiO_2_ ARHN is helpful to improve electromagnetic field as compared to NWs, the PEC measurement was performed to check the hot electron effect. Figure 7 shows the I-t curve measured under visible-light illumination. From Fig. 7, it is obvious that the photocurrent of Au/TiO_2_ ARHN is two times higher than Au/TiO_2_ NW. It means that the high plasmon electromagnetic field of Au/TiO_2_ ARHN results in a high hot-electron current. Therefore, we confirm that the design of TiO_2_ ARHN successfully provides a model for strengthening LSPR ability and demonstrates a remarkable enhancement on PEC performance.

**Figure 8:**
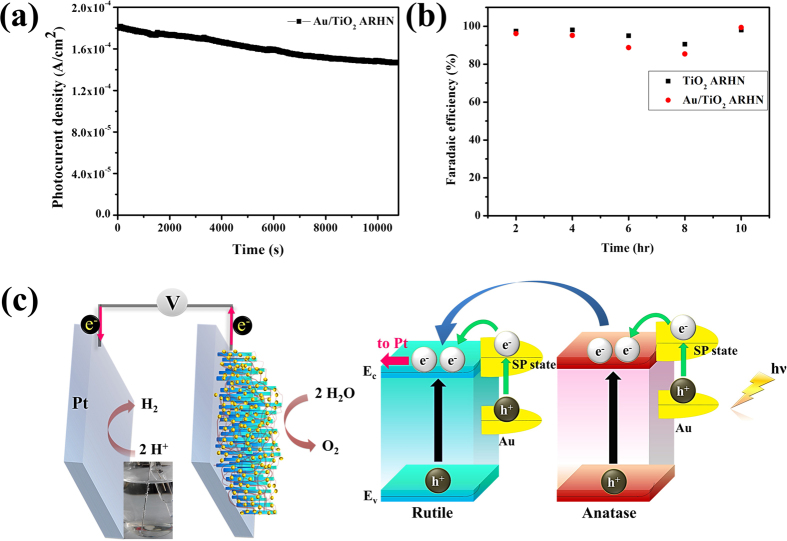
(**a**) Current―time curves of Au/TiO_2_ ARHN collected in 1 M NaOH at 0.23 V vs Ag/AgCl under 100 mW/cm^2^ for 3 h. (**b**) The calculated Faradaic efficiency for O_2_ gas evolution. (**c**) Charge transfer mechanism of Au/TiO_2_ ARHN under solar illumination.

In this study, we are further interested the effectiveness of density effect and size effect on the performance of photo electrochemical property. The related data and discussion were shown in Supplementary Information. In addition, the stability test and Faradaic efficiency were obtained. Under continuously illumination for 10800 seconds (equal to 3 hr), the photocurrent density was decrease from 1.8 × 10^−4^ A/cm^2^ to 1.5 × 10^−4^ A/cm^2^ in the case of Au/TiO_2_ ARHN, as shown in Fig. 8a. This photocurrent decay is similar to previous report^51^. We suggest it could be attributed to photo induced corrosion which competes with water oxidation reaction^51^. However, such a corrosion could be suppress by surface treatment of TiO_2_ nanostructure or use of sacrificial reagent/catalyst for longstanding application^51^. From Fig. 8b, the calculated Faradaic efficiency exceed 90% and 85% for TiO_2_ ARHN and Au/TiO_2_ ARHN, respectively. The high value of Faradaic efficiency of oxygen gas during 10 hr demonstrated that the photo generated current indeed utilized for water oxidation. Also, we could observe hydrogen gas from real picture as shown inset diagram in Fig. 8c. Therefore, we propose the electron transfer mechanism in Au/TiO_2_ ARHN system as shown in Fig. 8c. Under illumination, Au NPs absorb visible light, generating the energetic hot electrons from the process of SP excitation, and injecting them into the conduction band of the adjacent TiO_2_ (green arrow). Simultaneously, the UV light is absorbed by TiO_2_, producing a photo-excited electron and a hole (black arrow). The plasmon-induced electromagnetic field promotes the separation of photogenerated electrons and holes. Furthermore, as illustrated, the energy bands of anatase and rutile are different which provides a driving force to promote electron transfer from anatase to rutile (blue arrow). Finally, the electrons transferred to the cathode (Pt) react with H^+^ ions and produce H_2_ (pink arrow) whereas the holes present in the anode oxidize H_2_O and generate O_2_.

In conclusion, this work demonstrates a plasmon-induced effect on a designed 3D web architecture constructed from rutile TiO_2_ NWs, anatase TiO_2_ threads and Au NPs. Such a nanostructure was achieved for the first time through a simple and inexpensive hydrothermal procedure followed by calcination. The PEC performance tests, reveal that the photocurrent of Au/TiO_2_ ARHN was 4.5 times greater than that of the TiO_2_ NW photoelectrode. The observed optical properties and dark current measurements confirm that the excellent PEC performance of Au/TiO_2_ ARHN was due to three reasons: (1) the high surface area of TiO_2_ ARHN that increase the photoactive center, (2) the scattering effect in the TiO_2_ ARHN and the LSPR properties of Au NPs that enhanced the light harvest, (3) the strength coupling effect between Au NPs and TiO_2_ nanostructure that accelerated the charge transportation and separation. The mechanism of charge transportation in the Au/TiO_2_ ARHN was proposed based on our findings. Practical use of the Au/TiO_2_ ARHN was demonstrated to indicate their significant potential for use in photoelectric conversion system.

## Methods

### Materials

F:SnO_2_ (FTO) (1.5 cm × 3 cm, TEC-7, 7 ohm/sq., 2.2 mm thick) was used as the substrate for growth of the TiO_2_ film. All chemicals were used without further purification. Sodium hydroxide (NaOH, 99%), titanium tetrachloride (TiCl_4_, 99%), 2-butanone (C_4_H_8_O, >99%), hydrochloric acid (HCl, 12 M) and nitric acid (HNO_3_, 65%) were purchased from Merck. Tetrabutyl titanate (C_16_H_36_O_4_Ti, >97%) and ruthenium 535 bis-TBA (N719 dye) were obtained from Aldrich and Solaronix, respectively.

### Hydrothermal synthesis of TiO_2_ NWs on FTO substrates

First, FTO was cleaned by ultrasonic agitation in a mixture of ethanol, acetone and deionized water (volume ratio of 1:1:1) for 15 min. The FTO substrates were immersed in an aqueous of 0.5 M TiCl_4_ at 80 °C for 30 min, followed by heat treatment at 500 °C for 30 min to yield a thin TiO_2_ layer. The TiCl_4_-treated substrates were then suspended in a reagent solution containing 6 mL HCl, 6 mL 2-butanone and 0.6 mL tetrabutyl titanate in a Teflon vessel. The Teflon vessel was sealed in an autoclave and heated at 200 °C for 1.5 h. Further annealing at 500 °C for 30 min resulted in the growth of crystalline TiO_2_ NWs on FTO substrates^52^.

### Hydrothermal synthesis of TiO_2_ ARHN on FTO substrates

The TiO_2_ NW substrate (without calcination) was first treated with TiCl_4_ solution as mentioned above. A 200-nm-thick Ti layer was sputtered on the TiCl_4_-treated TiO_2_ NW using a magnetic sputter (K575X, Quorum Technologies). The substrates were then transferred to a Teflon vessel with the addition of a 5 M aqueous NaOH solution and were encapsulated in a stainless-steel autoclave. Then, the autoclave was heated at 80 °C for 30 min. After the low-temperature hydrothermal process, the substrate was rinsed with 0.1 M HNO_3_ followed by deionized water, and was finally calcined at 500 °C for 30 min to obtain hierarchical nanostructures.

### Synthesis of Au/TiO_2_ ARHN

A 5-nm Au layer was sputtered on the TiO_2_ NW and ARHN using a magnetic sputter. The Au deposited TiO_2_ were subsequently calcined at 500 °C for 1 h to obtain Au/TiO_2_ ARHN.

### Characterizations and measurements

The surface and cross-section morphologies of samples were examined by field-emission scanning electron microscopy (FE-SEM, Zeiss Ultraplus). The SigmaScan Pro 5 software was used to calculate particle size (300 particles were counted). The crystal structure was characterized by X-ray diffraction (XRD, PANalytical X’Pert Pro MRD) and Raman spectroscopy (Tokyo Instruments, INC). A UV/Vis spectrometer (Perkin Elmer/Lambda 900) was used to obtain the absorption spectra. The dye absorption/desorption experiment was performed by desorbing a dye-sensitized TiO_2_ electrode in a 0.1 M NaOH solution in 1:1 H_2_O/EtOH^53^. Subsequently, a UV–vis spectrometer was introduced to measure the absorbance of the desorbing solution. The electrochemical measurement was carried out using three-electrode system. TiO_2_ electrode with or without Au NPs was the working electrode; an Ag/AgCl (3 M KCl) electrode in saturated KCl was the reference electrode; the Pt plate was used as the counter electrode. All PEC cells were examined in 1 M NaOH solution with a PARSTAT 2263 Advanced Electrochemical System under illumination by Newport solar simulator with AM 1.5 G (100 mW/cm^2^). The incident photon-to-current conversion efficiency (IPCE) was measured with an action spectrum measurement setup (Peccell, PEC-S20).

## Additional Information

**How to cite this article**: Yen, Y.-C. *et al*. Plasmon-Enhanced Photocurrent using Gold Nanoparticles on a Three-Dimensional TiO_2_ Nanowire-Web Electrode. *Sci. Rep.* **7**, 42524; doi: 10.1038/srep42524 (2017).

**Publisher's note:** Springer Nature remains neutral with regard to jurisdictional claims in published maps and institutional affiliations.

## Supplementary Material

Supplementary Information

## Figures and Tables

**Figure 1:**
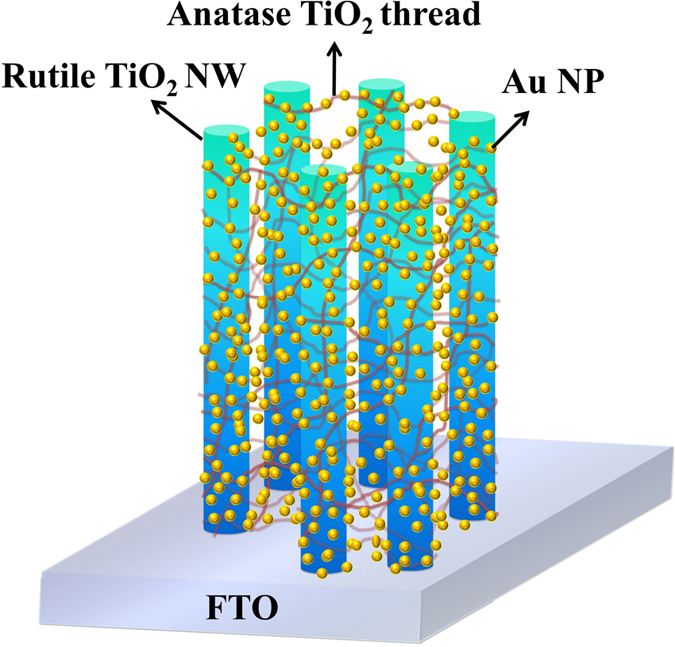
Schematic representation of Au/TiO_2_ ARHN on FTO substrates.
