# Supplementary material for: Plasmon-Enhanced Photocurrent using Gold Nanoparticles on a Three-Dimensional TiO2 Nanowire-Web Electrode
Source: Sci Rep. 2017 Feb 10;7:42524. doi: 10.1038/srep42524 (PMC5301249; doi:10.1038/srep42524)
Supplement: Supplementary Information [file srep42524-s1.pdf]

Supplementary Information

Plasmon-Enhanced Photocurrent using Gold Nanoparticles on a Three-Dimensional TiO_2 Nanowire-Web Electrode

*Yin-Cheng Yen, Jau-An Chen, Sheng Ou, Yi-Shin Chen and Kuan-Jiuh Lin**

Department of Chemistry, National Chung Hsing University, Taichung 40227, Taiwan, Republic of China

E-mail: kjlin@dragon.nchu.edu.tw

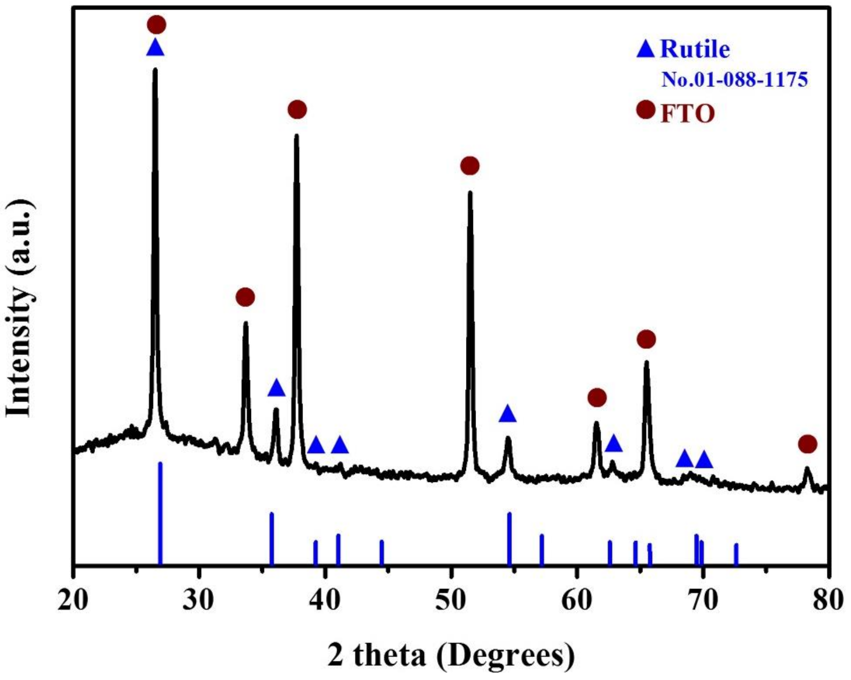

Figure S1. XRD pattern of TiO_2 NWs.

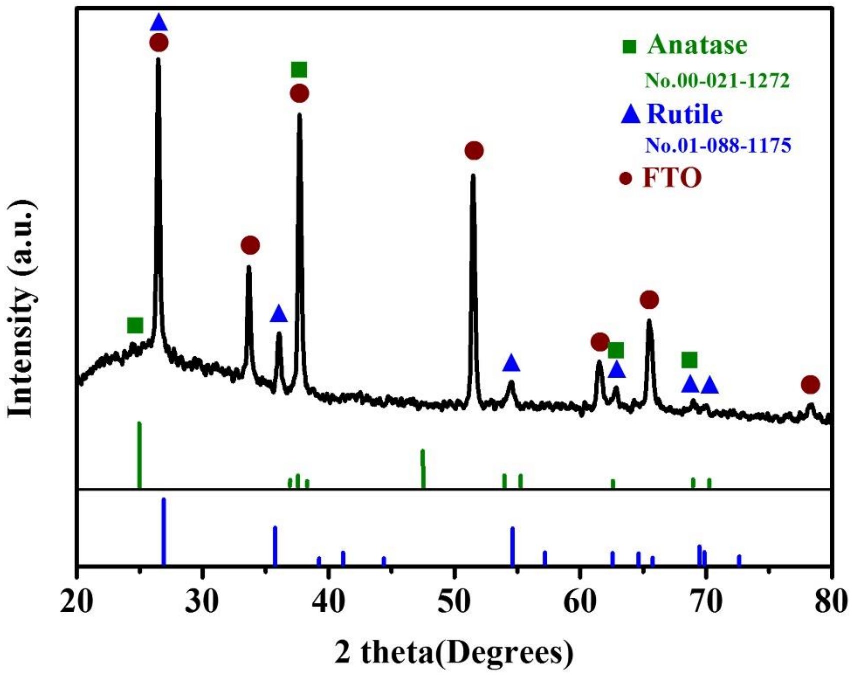

Figure S2: XRD pattern of TiO_2 ARHN.

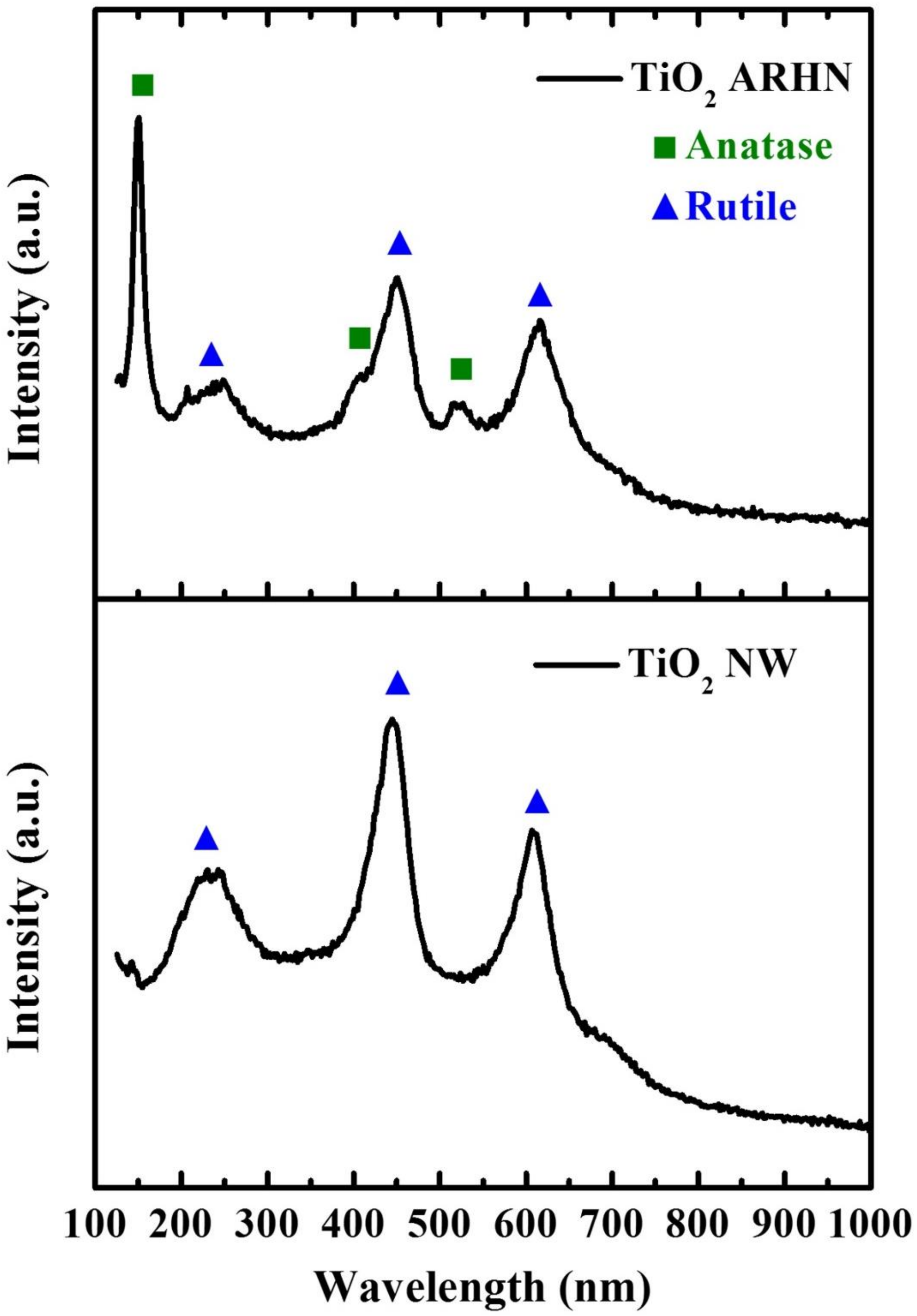

Figure S3. Raman spectra of TiO_2 NW and TiO_2 ARHN.

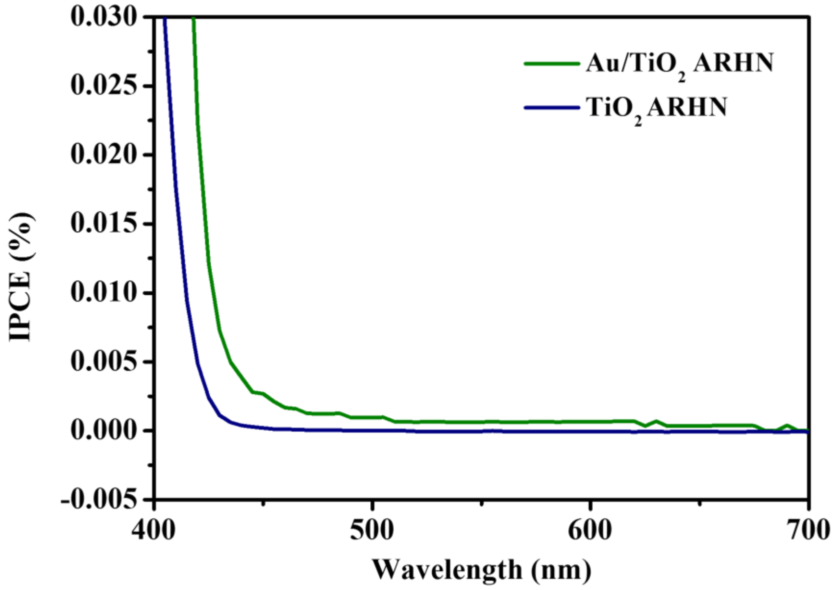

Figure S4. IPCE plots of TiO_2 ARHN and Au/TiO_2 ARHN in the range of 400 nm to 700 nm.

In this manuscript, Au NPs were deposited on TiO_2 nanostructure by magnetic sputtering an Au film and subsequently thermal annealing at high temperature. We did experiment found that, with change the thickness of Au film or the temperature of calcination, the density of Au NPs was change accompanied by a change in particle size and size distribution, as shown in Fig. S5 and S6. Therefore, it is difficult to investigate the influence of density of Au NPs on PEC's performance and make a conclusion accurately in our case due to the limitation on the experiment and the variable factors.

- The influence of calcination temperature

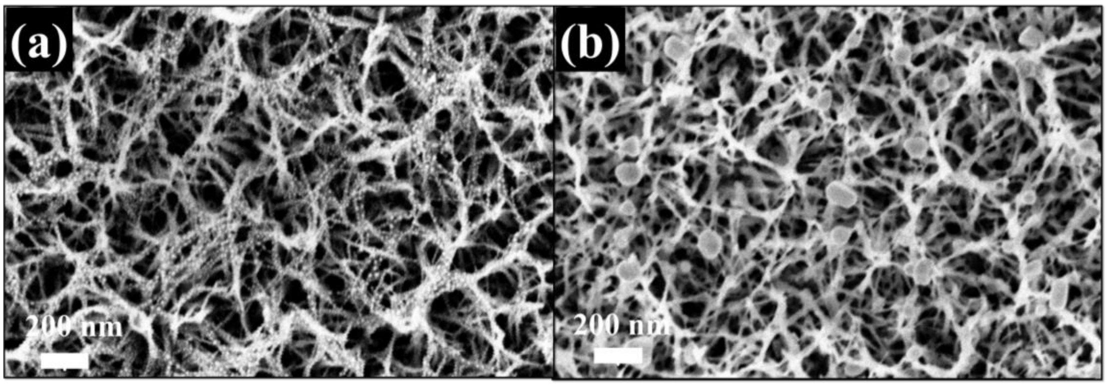

Figure S5. The SEM images of Au deposited TiO_2 calcined at (a) 400 °C and (b) 600 °C for 1 h respectively.

- The influence of thickness of Au film

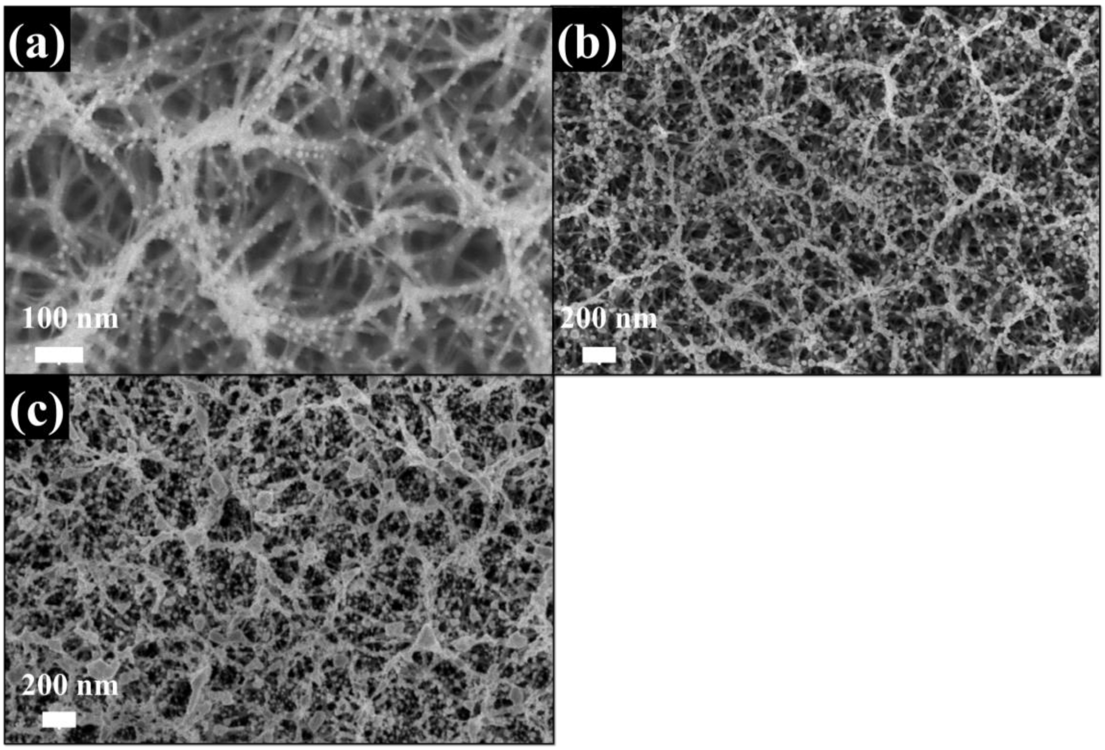

Figure S6. The SEM images of difference Au layer deposited TiO_2 calcined at 500 °C. (a) 1 nm (b) 7.5 nm and (c) 10 nm.
